# Transmembrane Amyloid-Related Proteins in CSF as Potential Biomarkers for Alzheimer’s Disease

**DOI:** 10.3389/fneur.2015.00125

**Published:** 2015-06-02

**Authors:** Inmaculada Lopez-Font, Inmaculada Cuchillo-Ibañez, Aitana Sogorb-Esteve, María-Salud García-Ayllón, Javier Sáez-Valero

**Affiliations:** ^1^Instituto de Neurociencias de Alicante, Universidad Miguel Hernández-CSIC, Sant Joan d’Alacant, Spain; ^2^Centro de Investigación Biomédica en Red sobre Enfermedades Neurodegenerativas (CIBERNED), Sant Joan d’Alacant, Spain; ^3^Unidad de Investigación, Fundación para el Fomento de la Investigación Sanitaria Biomédica de la Comunidad Valenciana (FISABIO), Hospital General Universitario de Elche, Elche, Spain

**Keywords:** Alzheimer’s disease, cerebrospinal fluid, BACE1, soluble amyloid precursor protein, presenilin-1, TACE

## Abstract

In the continuing search for new cerebrospinal fluid (CSF) biomarkers for Alzheimer’s disease (AD), reasonable candidates are the secretase enzymes involved in the processing of the amyloid precursor protein (APP), as well as the large proteolytic cleavage fragments sAPPα and sAPPβ. The enzymatic activities of some of these secretases, such as BACE1 and TACE, have been investigated as potential AD biomarkers, and it has been assumed that these activities present in human CSF result from the soluble truncated forms of the membrane-bound enzymes. However, we and others recently identified soluble forms of BACE1 and APP in CSF containing the intracellular domains, as well as the multi-pass transmembrane presenilin-1 (PS1) and other subunits of γ-secretase. We also review recent findings that suggest that most of these soluble transmembrane proteins could display self-association properties based on hydrophobic and/or ionic interactions leading to the formation of heteromeric complexes. The oligomerization state of these potential new biomarkers needs to be taken into consideration for assessing their real potential as CSF biomarkers for AD by adequate molecular tools.

## Introduction

Alzheimer’s disease (AD) is an age-related neurodegenerative disorder recognized as the most common cause of dementia among the elderly. The pathologic characteristics of AD are neurodegeneration and proteinaceous deposits, including extracellular plaques composed mostly of β-amyloid peptides (Aβ) and intracellular tangles of the microtubule-associated protein tau abnormally hyperphosphorylated (P-tau). Both pathological effectors, Aβ and P-tau, can be monitored in cerebrospinal fluid (CSF). In late-onset AD, concentrations of tau and P-tau in CSF are increased and probably reflect neuronal damage, but levels of Aβ peptides are decreased. These changes can be measured in CSF before the onset of any other symptoms, and, therefore, they can be used as a diagnostic marker for the disease [for a recent review, see Ref. (1)]. Although numerous laboratories have reported increased levels of P-tau and total tau (T-tau) in the CSF of AD patients, they are not specific, and also increase in other neuropathological disorders (2, 3). It is well recognized that Aβ peptides, and especially the Aβ42 species, are the most specific CSF biomarkers for AD.

According to the amyloid hypothesis, accumulation of Aβ in the brain, resulting from an imbalance between production and clearance, is the primary influence driving AD pathogenesis (4). The Aβ peptide is generated by processing a larger type I transmembrane spanning glycoprotein, the amyloid precursor protein (APP), through the successive action of proteolytic enzymes called secretases. Sequential processing of APP begins with either the action of α-secretase or β-secretase, followed by γ-secretase cleavage. When cleavage is carried out by β- and γ-secretase, the so-called amyloidogenic pathway, a 36–43 amino acid peptide is generated since γ-secretase acts on a domain with multiple potential cleavage sites (5). The Aβ40 peptide is the most common species, while the Aβ42 variant is the most amyloidogenic form of the peptide associated with AD progression. However, in the non-pathological condition, the majority of APP molecules are cleaved through the non-amyloidogenic pathway by the sequential action of α- and γ-secretases. α-Secretase cleaves APP within the Aβ domain, precluding the generation of the Aβ peptide [for a review, see Ref. (6)]. The existence in CSF of several shorter isoforms in addition to Aβ40 and Aβ42 has been explained by an alternative APP processing pathway involving concerted cleavages of APP by α- and β-secretase (7).

The predisposition for self-association of Aβ42 determines that while Aβ42 content is increased in the AD brain, its levels in CSF are decreased presumably due to its increasing deposition in brain tissue (2). In this context, with two dynamics playing out in opposite directions within the brain, increasing Aβ production and increasing deposition, the interpretation of CSF changes in Aβ levels in pre-symptomatic stages seems difficult. In fact, Jack et al. (8) proposed that Aβ-plaque biomarkers are dynamic early in the disease before the appearance of clinical symptoms, but have largely reached a plateau by the time clinical symptoms appear, determining that CSF Aβ does not change significantly over time in patients with AD. Moreover, in this context, it is difficult to anticipate, thus to evaluate, the outcomes expected from the CSF biochemical assessments of Aβ in AD subjects consequence of effective therapy with β- or γ-secretase inhibitors, potential disease-modifying therapeutics under development (9, 10).

In accordance with the mentioned challenges, there is a need to identify additional β-amyloid-related markers of AD. Reasonable candidates are proteins, such as secretases, involved in the pathological processing of APP, and the large proteolytic cleavage fragments sAPPα and sAPPβ. Since most of these secretases are transmembrane proteins, their assessments in CSF were not considered until recent years. The purpose of this article is to review recent evidence about the presence of secretase components in CSF and their potential as AD biomarkers. In addition, we summarize our recent findings about the presence of soluble full-length APP (sAPPf) in CSF and their oligomerization into heteromers. Our studies demonstrated that sAPP heteromers contribute to the estimation of sAPPα and sAPPβ levels, which needs to be taken into consideration for their assessment by ELISA. The suitability of applying adequate molecular tools for the assessment in CSF of hydrophobic proteins and soluble heteromeric aggregates is absolutely necessary to evaluate their potential as biomarkers.

## Soluble Full-Length and Heteromers of sAPP in CSF

**Figure 1:**
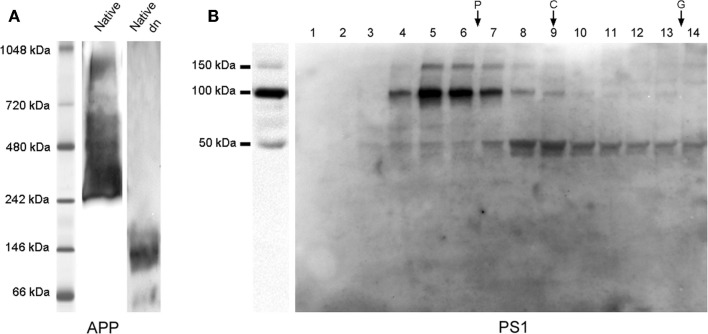
**sAPP and PS1 complexes in CSF**. **(A)** APP complexes from CSF samples analyzed by blue native-PAGE and resolved with a C-terminal antibody (from Sigma), confirmed the presence of APP complexes. A CSF sample denatured by boiling at 95°C for 5 min under fully reducing conditions (Dn) was also analyzed by blue native-PAGE to warrant the migration of the monomeric sAPP band. A similar banding profile was obtained with sAPPα and sAPPβ specific antibodies (not shown). See Ref. (16) for further details. **(B)** CSF samples were fractionated in 5–20% sucrose density gradients (left panel: same CSF sample prior fractionation). The fractions (collected from the top) were immunoblotted for PS1 with an anti N-terminal antibody (from Calbiochem). Enzymes of known sedimentation coefficient, β-galactosidase (G, 16.0S; ~540 kDa), catalase (C, 11.4S; ~232 kDa), and alkaline phosphatase (P, 6.1S; ~140–160 kDa) were used as internal markers. Incubation of blots with antibodies for the different γ-secretase subunits confirmed that APH1 and PEN2, but not nicastrin, are present in CSF as complexes (see Ref. (25) to complete information). In all analyses performed on PS1, denaturation before electrophoresis was conducted at 50°C.

The processing of APP begins with the action of either α-secretase or β-secretase, initiating mandatory pathways. The initial shedding by α-secretase or β-secretase releases large soluble proteolytic cleavage fragments of APP, sAPPα and sAPPβ, respectively, both present in human CSF (11, 12). Since amyloidogenic processing of APP is expected to be altered in the Alzheimer brain, both large sAPP fragments have been postulated as potential new AD biomarkers, but no consistent changes in CSF sAPPα and sAPPβ levels have been identified to date [see review by Perneczkyet al.(13)]. Interestingly, it has been suggested that full-length APP containing an intact cytoplasmic domain also exists as a soluble form (sAPPf) (14, 15). Recently, we confirmed that sAPPf is present in human CSF and demonstrated its contribution when estimating levels of large sAPP fragments (16). In consequence, the 6E10 antibody, a widely used anti-APP antibody that recognizes an epitope present in sAPPα and absent in sAPPβ, will detected not only sAPPα, but also sAPPf in CSF. Therefore, the use of 6E10 or similar antibodies in contraposition to pan-specific antibodies for the C-terminus of sAPPα should be considered as a contributing factor for contradictory findings between laboratories. Moreover, we have demonstrated that sAPPf co-exists in CSF with sAPPα and sAPPβ, and all forms are capable of assembling into heteromers [(16); see also Figure 1A]. The APP oligomerization status is particularly relevant, since most quantification of sAPPα and sAPPβ in CSF from AD subjects relies on ELISA determinations developed for monomeric species. Our data indicate that sAPP heteromers interfere with the measurement of sAPPα and sAPPβ in commercially available ELISA kits. Interestingly, an unexpected positive correlation has been consistently reported between both forms, indicating a similar shift for sAPPα and sAPPβ levels (17–20). Since the production of sAPPβ should be inversely proportional to that of sAPPα, this is an unexpected finding that we attributed, at least in part, to the existence of sAPPα/sAPPβ heteromers. In this context, early studies assessing sAPPα and sAPPβ levels by Western blot failed to demonstrate this positive correspondence (21). The assessment of sAPPα/sAPPβ levels is also of interest to monitor the biochemical effect of drugs targeting Aβ in clinical trials (22), particularly for β-/γ-secretase inhibitors since discouraging reports question this therapeutic strategy, even the amyloid cascade hypothesis (23). In this regard, β-secretase inhibition resulted in sAPPβ significant decrease, but also in increased concentration of sAPPα (24), suggesting that inhibition of β-secretase in humans resulted in a compensatory increase in non-amyloidogenic APP cleavage. The simultaneous determination of sAPPα and sAPPβ in CSF by protocols that prevents underestimation by heteromeric association is mandatory.

In conclusion, an optimal approach to quantify sAPPα and sAPPβ in CSF has been based on ELISA determinations, but the presence of heteromeric complexes of sAPP obligate adjusting protocols. Moreover, the characterization of a soluble transmembrane protein might be hindered by the difficulty in distinguishing it from the truncated species generated by cleavage of the transmembrane protein. The existence of different sAPP isoforms, generated from alternative exon splicing (26), adds complexity to the determination of sAPP as CSF biomarkers, but needs to be taken into consideration since large species of sAPP, which should correspond to APP751/770, appeared to increase AD CSF (16). Analysis of sAPPf splicing isoforms may be of particular interest and needs to be more specifically addressed. More research is needed to design an appropriate strategy and assays for CSF sAPP. The validation of sAPP as a CSF biomarker may be of particular interest for assessing the effect of clinical trials based on β-secretase inhibition, where a decrease in newly generated sAPPβ is expected, but with an unclear effect on newly generated sAPPα (27).

## β-Secretase and TACE/α-Secretase Activities in CSF

The major neuronal β-secretase has been identified as beta-site APP cleaving enzyme 1 [BACE1; (28)], though other proteases such as BACE2 and cathepsins might be involved as well (29, 30). Interestingly, both BACE1 protein and activity levels can be measured in CSF (31), but, to date, accurate determination of BACE1 remains a great challenge and there is no consensus as to whether its levels are consistently affected in CSF as dementia progresses (32). Most published results suggest that BACE1 activity increases in AD, preferentially in MCI cases with prodromal AD (31, 33, 34). However, such biochemical analysis often relies on APP fluorogenic substrates with modified APP β-cleavage sites, whose discrimination between BACE1 from other β-secretase enzymes like BACE2 and cathepsins is unclear [for a review, see Ref. (32)]. Currently, it is not known whether BACE1 activity reflects BACE1 protein content since it correlates poorly (33), and it is also unknown if the values measured are due to full-length BACE1 or a truncated form. Mature BACE1 holoprotein contains a single transmembrane domain and a short intracellular C-terminal (28). Membrane-bound BACE1 can be partly cleaved within its extracellular domain to generate soluble BACE1 for secretion (35, 36). Accordingly, it has been assumed that the BACE1 present in CSF is a truncated soluble form of the originally membrane-bound BACE1 missing the transmembrane and intracellular domains (37). Indeed, some studies failed to demonstrate the presence of BACE1 containing the C-terminal domain in human CSF and plasma (38, 39), but others detected immunoreactivity with BACE1 C-terminal antibodies (25, 31, 33), suggesting that full-length BACE1 exists in this fluid. The presence of full-length BACE1, together with its truncated form, has also been demonstrated in conditioned media from cultured neurons (40). The presence in CSF of an immature form of BACE1 protein, poorly active or inactive, has also been suggested (33). Future work will be required to elucidate if both the full-length and truncated BACE1 account for β-secretase in CSF.

Furthermore, similarly to APP, BACE1 occurred as a dimer in human brain tissue (41, 42). Therefore, we cannot discard the occurrence of BACE1 forming complexes in CSF, which needs to be taken into consideration, especially for the attempts to develop BACE1 ELISA assays (33, 43). In conclusion, extensive work remains to be accomplished to reinforce the interest of using CSF BACE1 levels and activity as AD biomarkers.

Regarding α-secretase, at least three members of the ADAM (a disintegrin and metalloproteinase) family, ADAM10, ADAM17 (TACE), and ADAM9 have been proposed as α-secretases (44), and other ADAM family members, such as ADAM8, has also demonstrated efficiency in cleavage of APP (45). Evidence indicates that ADAM10, but not ADAM9 or ADAM17, is the enzyme acting as a physiologically relevant constitutive α-secretase *in vivo* (46, 47). To our knowledge, the occurrence of ADAM-10/α-secretase activity in either CSF or plasma has not been reported to date, and ADAM10 has so far only been found in platelets (48) and other blood cells (49). However, elevated activity levels for ADAM17/TACE activity have been found in both CSF (50) and plasma (51, 52) from subjects with AD. TACE releases several transmembrane proteins into soluble forms, including APP, but also tumor necrosis factor α (TNFα) receptors (53). The synthetic peptide used for TACE enzymatic activity assays in CSF and plasma consists of a TACE-sensitive TNF sequence surrounding the TACE-specific cleaving site (50); thus, it constitutes a substrate favorable for TACE compared to ADAM10. α-Secretase accurately refers to the activity targeting APP and generating sAPPα; nonetheless, the general requirements for secretase cleavage are not strict and we cannot exclude the possibility that other enzymes, including ADAM10, may cleave peptides in human CSF and plasma. The presence in CSF of other ADAM family members, including ADAM10, deserves study.

Moreover, ADAM proteases, similarly to BACE1, are type I transmembrane proteins, but also include secreted isoforms (44). Indeed, ADAM10 and ADAM17 have been shown to be secreted outside the cells in exosomes (54). Thus, the occurrence of TACE activity in CSF and plasma has been attributed to soluble isoforms shedding from cell membranes after the cleavage of TNFα and the TNF receptors. Nonetheless, TACE protein has only been studied in plasma by Western blot using an anti-TACE polyclonal antibody (52), but not by the combination of N- and C-terminal antibodies allowing characterization of the full-length and truncated forms. Again, a parallel study of protein and enzyme activity is pending in order to define the most sensitive molecular tools necessary for using ADAM as CSF biomarkers.

## Presenilin-1 and Other γ-Secretase Components are Present in CSF

γ-Secretase is an intramembrane protease complex composed of presenilin-1 (PS1), nicastrin, APH1 (anterior pharynx-defective 1), and PEN2 (presenilin enhancer 2) (55). Since most of the γ-secretase components contain several transmembrane domains, their presence in CSF was not assessed until recently. PS1 is a transmembrane aspartyl protease and the catalytic subunit of γ-secretase, and it is known to undergo endoproteolytic cleavage as part of its maturation, generating N- and C-terminal fragments (NTF and CTF) (56), with six- and three-transmembrane domains, respectively (57). APH1 also displays seven-transmembrane domains and PEN2 two transmembrane domains; only nicastrin contains a single transmembrane domain [for a review, see Ref. (58)]. Previously, the presence of soluble CTF–PS1 was reported in the media from cultured neurons (59). Recently, we demonstrated the presence of 100–150-kDa heteromeric complexes in CSF, composed of NTF and CTF PS1 [(25); see also Figure 1B]. The presence of the NTF and 20-kDa CTF fragments was only clearly detectable in *post-mortem* CSF, where artifacts are likely to appear. APH1 and PEN2, but not nicastrin, co-exist within these CSF–PS1 complexes. We were unable to detect γ-secretase activity in human CSF, and presumed that CSF–PS1 complexes may result from non-specific aggregation of these transmembrane proteins with large numbers of hydrophobic regions. PS1 aggregates have previously been described as temperature-sensitive (60); similarly, CSF–PS1 complexes are only detectable when denaturation before electrophoresis is conducted at 50°C (15 min). Thus, analysis performed with samples denatured at 98°C can underestimate and fail to detect PS1 complexes. Ultracentrifugation in sucrose density gradients confirmed the existence of stable complexes of 100–150-kDa, but also showed that large complexes, which sediment in regions closer to 200 and 250 kDa, are unstable during electrophoresis under denaturing conditions. Interestingly, when we assessed whether CSF–PS1 levels are altered in AD, ventricular *post-mortem* samples (disease at term) display higher levels of PS1 than those present in non-demented control cases, particularly the stable complexes resolved by sucrose density gradients. Lumbar CSF samples from probable AD cases (early stages of the disease) display only significant differences in the proportion of the PS1 stable complex, but not in total levels (25). The amount of PS1 stable complexes correlates with Aβ42. Our results suggest that the early and more significant phenomenon is the change in the dynamics of the assembly of PS1 complexes. The change in the proportion of stable complexes appears as a better marker for discriminating pathological samples than the estimation of total PS1 protein levels. Further characterization of CSF–PS1 complexes has yet to be conducted in order to define the appropriate methodological approach for evaluating their feasibility as a potential new AD biomarker, as well evaluation of its diagnostic performance in comparison with existing biomarkers such as Aβ.

## Conclusion

Because CSF is in direct contact with the extracellular space of the central nervous system, biochemical changes in the brain could potentially be reflected in CSF. It is expected that potential AD biomarkers involved in AD pathogenesis will mirror AD progression. However, to date, no single biomarker has reached expectations. Several models of CSF secretion have been proposed (61–63), but the relationship with protein content and cellular origin of CSF protein composition remains unclear. Moreover, increasing evidence indicates the occurrence of soluble full-length membrane proteins in CSF. The mechanisms by which these membrane-bound proteins reached the CSF are unknown. Active secretion is unlikely, and it is still unclear if passive release from brain cells or neuronal death may be major contributing factors, as recently observed for BACE1 (43). Most of these forms could display self-association properties based on hydrophobic and/or ionic interactions, resulting in the formation of complexes. Indeed, proteins like presenilins, with large numbers of hydrophobic regions, may be highly unstable in CSF, and spontaneously form complexes. The occurrence of different types of protein complexes in CSF, forming heterogeneous components, should be considered to accurately determine their levels. In this sense, the presence in CSF of soluble oligomers, normally associated with protein-misfolding diseases, has been suggested for multiple sclerosis patients (64).

Our understanding of the potential roles for APP, BACE1, ADAM proteins, PS1, and other related proteins in CSF is lacking, but of interest in order to design adequate quantification strategies to assess their real potential as biomarkers for AD. Ultimately, it is anticipated that a combination of CSF biomarkers might serve for early diagnosis, but also for assessing disease progression and especially the efficiency of secretase inhibitors during the course of clinical trials. In this review, we presented evidence that most of the proteins related with APP processing are measurable in CSF. More investigation should focus on the possibility of monitoring soluble forms of APP and secretase components, and to evaluate the progress and feasibility of developing molecular tools for these potential new CSF biomarkers for AD.

## Conflict of Interest Statement

The authors declare that the research was conducted in the absence of any commercial or financial relationships that could be construed as a potential conflict of interest.
